# Cardioprotective Effect of Glycyrrhizin on Myocardial Remodeling in Diabetic Rats

**DOI:** 10.3390/biom11040569

**Published:** 2021-04-13

**Authors:** Vikram Thakur, Narah Alcoreza, Monica Delgado, Binata Joddar, Munmun Chattopadhyay

**Affiliations:** 1 Center of Emphasis in Diabetes and Metabolism, Department of Molecular and Translational Medicine, Paul L. Foster School of Medicine, Texas Tech University Health Sciences Center, El Paso, TX 79905, USA; vikram.thakur@ttuhsc.edu; 2 Graduate School of Biomedical Sciences, Texas Tech University Health Sciences Center, El Paso, TX 79905, USA; nalcorez@gmail.com; 3 Inspired Materials & Stem-Cell Based Tissue Engineering Laboratory (IMSTEL), Department of Metallurgical, Materials and Biomedical Engineering, The University of Texas at El Paso, El Paso, TX 79968, USA; mdelgado7@miners.utep.edu (M.D.); bjoddar@utep.edu (B.J.)

**Keywords:** glycyrrhizin, diabetic, cardiac atrophy, inflammation, CXCR4

## Abstract

Myocardial fibrosis is one of the major complications of long-term diabetes. Hyperglycemia induced cardiomyocyte atrophy is a frequent pathophysiological indicator of diabetic heart. The objective of this study was to investigate the cardioprotective effect of glycyrrhizin (GLC) on myocardial damage in diabetic rats and assess the anti-inflammatory and anti-fibrotic effect of GLC. Our study demonstrates that hyperglycemia can elevate cardiac atrophy in diabetic animals. Type 2 diabetic fatty and the lean control rats were evaluated for cardiac damage and inflammation at 8–12 weeks after the development of diabetes. Western blot and immunohistochemical studies revealed that gap junction protein connexin-43 (CX43), cardiac injury marker troponin I, cardiac muscle specific voltage gated sodium channel Na_V_1.5 were significantly altered in the diabetic heart. Furthermore, oxidative stress mediator receptor for advanced glycation end-products (RAGE), as well as inflammatory mediator phospho-p38 MAPK and chemokine receptor CXCR4 were increased in the diabetic heart whereas the expression of nuclear factor erythroid-2-related factor 2 (Nrf2), the antioxidant proteins that protect against oxidative damage was reduced. We also observed an increase in the expression of the pleiotropic cytokine, transforming growth factor beta (TGF-β) in the diabetic heart. GLC treatment exhibited a decrease in the expression of phospho-p38 MAPK, RAGE, Na_V_1.5 and TGF-β and it also altered the expression of CX43, CXCR4, Nrf2 and troponin I. These observations suggest that GLC possesses cardioprotective effects in diabetic cardiac atrophy and that these effects could be mediated through activation of Nrf2 and inhibition of CXCR4/SDF1 as well as TGF-β/p38MAPK signaling pathway.

## 1. Introduction

Chronic hyperglycemia leads to cardiovascular impairment. Increasing evidence suggests that myocardial dysfunction is vastly prevalent in diabetic patients [1,2]. Prolonged exposure to high glucose due to diabetes can affect macro and microvascular systems causing cardiovascular complications [3,4]. At present, 463 million people are living with diabetes worldwide and it is projected to rise to 700 million by the year 2045. Approximately 11.3% deaths worldwide are attributed to diabetes and its complications [5] and cardiovascular diseases are one of the major complications, as well as a major cause of mortality in diabetic patients [6]. Damage to the cardiomyocytes, the cells that are responsible for generating contractile forces in the heart could lead to the development of diabetic cardiomyopathy [7]. A number of mechanisms prompting cardiomyocyte death have been projected, including inflammation, oxidative stress, glucose mediated toxicity and mitochondrial damage [8]. However, the pathogenesis of cardiac atrophy is not completely understood. Cardiac cells or cardiomyocytes may experience very early changes under hyperglycemic condition that may lead to release of crucial inflammatory mediators such as cytokines and chemokines [9,10]. Circulating high levels of glucose could lead to altered cardiomyocyte signaling which may in turn lead to oxidative stress, fibrosis and ultimately to myocyte cell death [11].

Although hyperglycemia is the major influence in myocardial dysfunction, alterations in glucose levels may help resolve the glucose mediated toxicity. The effect of hyperglycemic insult could also be rescued by inhibiting the inflammation cascade by therapeutic approaches [12,13,14]. Uncontrolled long-term hyperglycemia could effectuate cardiac fibrosis, the pathogenesis of which is complex. A number of factors or pathways could be involved in this process, including increase in oxidative stress markers such as RAGE, cardio-fibroblast proliferation, increased expression of inflammatory cytokines such as TGFβ, chemokine receptors such as CXCR4 and alterations in phospho-p38 MAPK expression [15,16,17]. An increasing amount of evidence suggest that inflammation may play an important role in the pathogenic processes in the cardiovascular disease [18,19]. A number of studies have exhibited that circulating troponin expression is higher in diabetic patients with cardiovascular disease compared to patients without diabetes, thereby indicating that it may play an important role as a marker of cardiac inflammation in hyperglycemia [20,21].

Glycyrrhizin (GLC), a bioactive triterpenoid saponin, is a key active component of licorice root with anti-inflammatory and antioxidant properties and has been demonstrated to alleviate hepatotoxicity, gastritis and other ailments [22,23,24,25]. A recent study revealed cardio-protective effect of GLC in isoproterenol-induced myocardial ischemia injury in rats [26]. Previous studies have also shown that GLC alleviates hepatocyte apoptosis and liver fibrosis [22,23]. In a prior study, our group established that GLC treatment was effective in alleviating pain in diabetic neuropathy [27]. Another study revealed the neuroprotective effect of GLC in the cerebral ischemia/reperfusion injury mediated inflammation and oxidative stress in the rat brain [28].

Presently, therapeutic opportunities for hyperglycemia mediated cardiac damage are not adequate and the main focus to improve overall cardiac function is via managing hyperglycemia and use of lipid-lowering medications [29,30]. It is imperative, from a number of studies, that strategies to reduce inflammation or oxidative stress to improve cardiac function in diabetes are critical [31,32]. Therefore, anti-inflammatory agents may prove to be efficient prospective therapeutic targets in preventing development and progression of cardiovascular impairment. Our study exhibited hyperglycemia induced cardiomyocyte atrophy in both in vitro and in vivo studies. Thus, we examined the changes in hyperglycemia-induced cardiomyocytes and the heart tissue of type 2 ZDF diabetic rats and investigated the possible role of inflammatory mediators to understand the mechanism involved in the pathogenesis of cardiovascular atrophy in hyperglycemic condition and examined how GLC could be used as a therapeutic approach to facilitate its prevention.

## 2. Materials and Methods

### 2.1. Experimental Animals

Spontaneous type 2 diabetic model of Zucker diabetic fatty (ZDF, Charles River, Wilmington, MA, USA) rats and lean control rats were used in this study. The animals were housed in pairs in the institutional laboratory animal SPF facility with the recommended light/dark cycle, humidity and temperature. Diabetic ZDF rats were given Purina #5008 lab diet and water, ad libitum during the entire experimental timeline. Nine to 10 weeks old ZDF rats with blood glucose level around 300 mg/dl were included in the study along with age-match controls. OneTouch Ultra glucose meter (LifeScan, Inc; Milpitas, CA, USA) was used to measure blood glucose levels once a week. Experiments were carried out according to the approved institutional animal care and use protocol (IACUC, TTUHSC El Paso, TX, USA). All experiments were performed following ARRIVE guidelines [33].

### 2.2. Treatment and Tissue Collection

Fourteen weeks old diabetic animals were injected with Glycyrrhizin (GLC) at a dose of 50 mg/kg per day I.P for 5 days a week for 4 weeks [27]. Animals were divided into 3 groups: lean control, diabetic only with vehicle treatment, diabetic with GLC treatment. Each group was comprised of 8–10 animals. At the end of the four weeks treatment, animals were euthanized for tissue collection according to the AVMA guidelines for Euthanasia to collect heart tissue samples as proposed in the approved IACUC policy. The treatment with GLC did not change the blood glucose levels or weight of the diabetic animals (see Supplementary Materials Figure S2 from published article by the first and senior authors as the cardiac tissues from the same animals were used) [27]. The whole heart tissues were harvested in dry ice after ice-cold 0.9% saline perfusion and immediately stored at −80 °C for further use. A low dose of GLC (50 mg/kg/day) was used, in this study, to reduce any systemic complications.

### 2.3. Western Blot

Fresh heart tissues of rats from all 3 groups were harvested and processed for western blot as described in our previous work [34]. The primary antibodies for RAGE, CX43, TGFβ, GAPDH (Cell Signaling, Danvers, MA, USA), CXCR4, Nrf2, ET-1 (Thermo Fisher Scientific, Waltham, MA, USA), phospho-p38 (Santa Cruz Biotechnology Inc., Dallas, TX, USA), Na_V_1.5 (NIH NeuroMab, Davis, CA, USA) and secondary antibody anti-rabbit IgG or anti-mouse IgG (1:5000 Amersham, Piscataway, NJ, USA) were used according to the protocol and imaged with ECL (Pierce, Rockford, IL, USA). β-actin (1:2000; MilliporeSigma, St. Louis, MO, USA) was used as a loading control and the data were normalized with the respective level of β-actin using an image analysis software (ChemiDoc XRS System, Bio-Rad Laboratories, Hercules, CA, USA) to determine the intensity of each band (*n* = 3–5 animals per group), the data were further analyzed to assess the percent of control.

### 2.4. Histopathological Analysis

Part of the ventricular heart tissue was embedded in OCT embedding medium and the tissue was cut in 10 µm sections and fixed in 4% paraformaldehyde and washed in running water for 5 min. The sections were subsequently stained in Harris modified Hematoxylin solution with 5 min, followed by running water wash until clear. The sections were then immersed in Gomori’s trichrome stain for 15 min and differentiated in 1% acetic acid for 1 min. The sections were dehydrated in two changes of 95% and 100% alcohol solutions and were then cleared in three changes of xylene (5 min each) followed by mounting of coverslips onto the tissue with Permount mounting medium. Gomori’s Trichrome stained sections were obtained using a Nikon Eclipse Ni-E microscope (Nikon Instruments Inc., Melville, NY, USA) to evaluate collagen deposition.

### 2.5. Collagen Deposition Quantification

Gomori’s Trichrome stained cardiac tissue sections were measured for collagen density and were compared among naïve control, diabetic alone, diabetic with GLC treatment groups to identify the effects of the GLC treatment on the collagen deposition in the cardiac ventricular muscle. The ImageJ image analysis software was used to measure the intensity of the Trichrome stained fibers as described by the authors earlier [35]. The images were acquired with 20X objective using a Nikon Eclipse Ni-E microscope. An investigator blinded to the treatment group evaluated the tissue sections from 3 different areas for each animal.

### 2.6. Immunohistochemistry and Immuno-Histochemical Analysis

At the end of the treatment regimen, the whole heart was collected, fixed with 4% paraformaldehyde overnight, followed by cryoprotection with 30% sucrose in phosphate buffered saline (PBS) for 1–2 days. The heart was incised into smaller pieces, embedded in OCT embedding solution and stored at −80 °C. The blocks were cut in 10 µm thick sections and placed on specific gelatin-coated slides. The sections were fixed with 4% paraformaldehyde once more for 30 min, washed with PBS 3 times, incubated with blocking solution (PBS with 1% NGS and 0.3% Triton X-100) for 60 min. The sections were washed once and incubated with the primary antibody anti-CX43 (1:400; Cell Signaling, Danvers, MA, USA) or Troponin-I (1:500; Thermo-Fisher Scientific, Waltham, MA, USA), for overnight at 4 °C followed by 3 washes. Alexa Fluor 594 goat anti-rabbit IgG and 488 goat anti-mouse IgG (1:2000; Thermo-Fisher Scientific, Waltham, MA, USA) used as secondary antibodies and were incubated for one hour at room temperature. Slides were stained with DAPI following three washes and mounted in Fluoromount G (Electron Microscopy Sciences, Fort Washington, PA, USA).

Troponin-I (TnI) stained cardiac tissue sections were measured for changes in troponin intensity and were compared among naïve control, diabetic alone, diabetic with GLC treatment groups to identify the effects of the GLC treatment on the TnI in the cardiac ventricular muscle. The Nis Elements software (Nikon Instruments Inc., Melville, NY, USA) was used to measure the intensity of the TnI stained images. An investigator blinded to the treatment group evaluated the tissue sections from 3 animals per group and 3–5 different areas for each animal.

### 2.7. Cell Culture and Treatment

AC16 human cardiomyocyte (CM) cell line (Millipore cat no. SCC109) was cultured in Dulbecco’s Modified Eagle’s Medium/Nutrient Mixture F-12 Ham (DME/F-12) supplemented with 10% fetal bovine serum (FBS). This cell line was initially obtained from the primary human ventricular cardiac tissue cells with SV40 transformed fused with uridine auxotroph human fibroblasts, deficient of mitochondrial DNA. The cells were seeded overnight in culture plates at 50% confluency. When the cells had reached 70–80% confluency, the wells were divided evenly into control and experimental groups (*n* = 2 per group). The control group had only cardiomyocytes and no intervention. First, experimental group cardiomyocytes were exposed to CXCR4 agonist stromal cell-derived factor 1 (SDF1; also known as CXCL12) at 50 ng/mL to simulate the receptor CXCR4 expression. A group of cardiomyocytes with SDF1 were exposed to 50 µm of GLC. In the second experiment, a group of AC16 cardiomyocyte cells were treated 25 mM of glucose overnight and a group of hyperglycemic cells were treated with 20 µm of CXCR4 antagonist AMD3100 and another group of hyperglycemic cells were treated with 50 µm of GLC to confirm anti-inflammatory effect of GLC. At the end of the 24 h time point, cells were harvested, protein extraction procedures and analysis were performed as described above.

### 2.8. Statistical Analysis

Control, diabetic and treated diabetic rats were indiscriminately assigned to their respective experimental groups. Samples were evaluated randomly. Analysis of variance (ANOVA) was conducted was used for the group comparisons and Bonferroni’s multiple comparison tests in post hoc analysis was followed. All the statistical analysis was accomplished using SPSS software (Systat version 13.0, SPSS Inc., Chicago, IL, USA) and a *p*-value < 0.05 was considered as a statistically significant difference.

## 3. Results

### 3.1. Type 2 Diabetic Animals Treated with GLC Exhibited a Significant Change in Collagen Deposition without Any Change in Blood Glucose Levels

**Figure 1:**
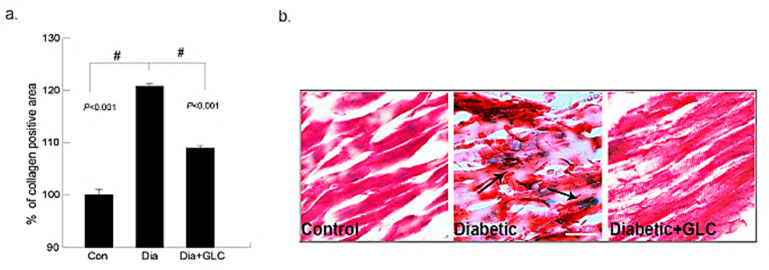
Ventricular heart tissues of type 2 diabetic animals exhibited a significant increase in collagen deposition which was ameliorated after GLC treatment. Changes in collagen deposition in ZDF type 2 diabetic, diabetic with GLC treatment and lean control rats: (**a**) the measurement of collagen positive area; percent of control calculated from the positive trichrome staining (Means ± SE, *n* = 5); significant level: # *p* < 0.001; (**b**) histopathological assessment of Gomori’s trichrome stain in control, diabetic and diabetic with GLC groups, arrows indicating collagen deposition and fibrosis. Representative images of Gomori’s trichrome staining of ventricular heart tissues in Con, ZDF diabetic and ZDF diabetic with GLC treated rats (Scale bar = 50 µm).

The deposition of collagen in the early phase of cardiac fibrosis in the ZDF, lean and ZDF rats treated with GLC was analyzed by histopathological examination of Gomori’s trichrome stain. The collagen (blueish green) deposition in the heart was significantly higher in ZDF diabetic rats at eighteen weeks of age than that of the age-matched lean controls, as shown in Figure 1. Furthermore, the fibrosis in the cardiac muscles of ZDF rats was observed. The ZDF animals treated with GLC showed a significant reduction in the collagen (blueish green) deposition in the heart relative to the ZDF rats without treatment (*p* < 0.01). Overall, the Gomori’s trichrome stain revealed that diabetes can promote increased collagen deposition in the cardiac tissues as shown in the Figure 1 (collagen deposition stained blue, cytoplasm and muscle fibers stained red). The treatment with GLC did not change blood glucose levels in diabetic animals (Supplementary Materials Figure S2).

### 3.2. GLC Altered Chemokine Receptor CXCR4 and Connexin-43 Expression in Cardiac Tissue of Type 2 Diabetic Rats

**Figure 2:**
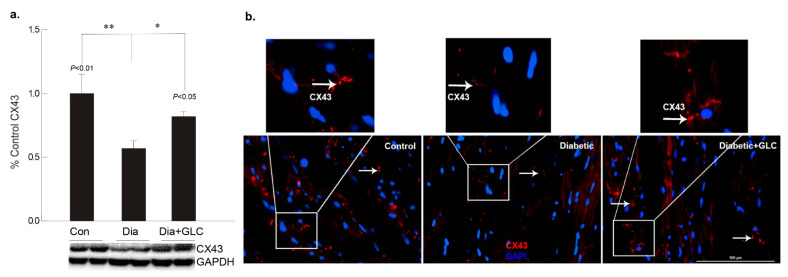
GLC alleviated the expression of gap junction protein connexin-43 in cardiac tissue of Type 2 diabetic rats. Gap junction protein connexin43 (Cx43) expression was assessed by Western blot analysis as well as by immunohistochemistry in ZDF type 2 diabetic, diabetic with GLC treatment and lean control rats. (**a**) Western blot analysis demonstrated a decrease in CX43 expression in the ventricular cardiac tissue of diabetic animals was noted compared to the lean control animals, ** *p* < 0.01; GLC treated cardiac tissue exhibited restoration of CX43 expression in the diabetic with GLC treated group compared to the diabetic only group * *p* < 0.05. (**b**) Immunohistochemical analysis also exhibited reduced CX43 expression in diabetic animals whereas GLC treated animals displayed improved CX43 expression; arrows indicate CX43 positive staining in red (Scale bar = 100 µm).

**Figure 3:**
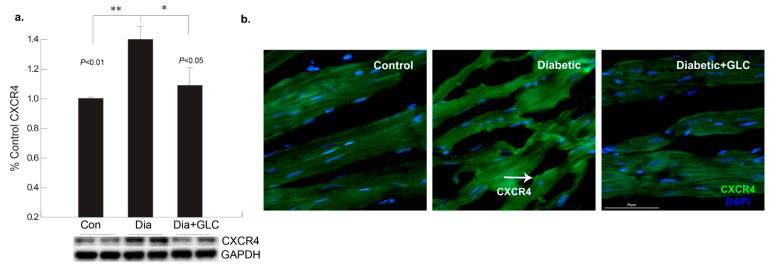
Increase in chemokine receptor CXCR4 expression in the diabetic heart tissue was reduced after 4 weeks of GLC treatment. (**a**) Chemokine receptor CXCR4 expression was increased significantly the ventricular tissue of ZDF rats compared to lean control animals as demonstrated by Western blot analysis (** *p* < 0.01) and treatment with GLC revealed alteration in the expression of CXCR4 (* *p* < 0.05). (**b**) Immunohistochemical illustrations further confirmed the decrease in the CXCR4 expression by GLC treatment in the diabetic animals compared to diabetic group; arrow indicates CXCR4 positive staining (Scale bar = 50 µm).

To better understand the regulation of cell adhesion in diabetic condition, gap junction protein connexin43 (Cx43) was assessed. Western blot analysis demonstrated a reduction of CX43 expression in the cardiac tissue of diabetic animals compared to the lean control animals (Figure 2a); whereas GLC treated animals exhibited restoration of CX43 expression which was confirmed by immune-histochemical studies (Figure 2b). To understand the underlying mechanisms and the role of inflammatory mediators in cardiac fibrosis, the CXC chemokine receptor 4 (CXCR4), a G protein-coupled receptor (GPCR) was evaluated. Western blot analysis of the ventricular tissue of ZDF rats demonstrated a significant increase in the CXCR4 expression compared to lean control animals and treatment with GLC also led to an alteration in the expression of CXCR4 (Figure 3a), which further confirmed its possible role in perpetuation of inflammation. The decrease in the CXCR4 expression by GLC treatment as confirmed by Western blot and Immunohistochemistry suggests an anti-inflammatory effect in the diabetic cardiac atrophy (Figure 3a,b).

### 3.3. ZDF Rats Exhibited Increased Troponin-I in the Cardiac Tissue which Was Alleviated with GLC Treatment

**Figure 4:**
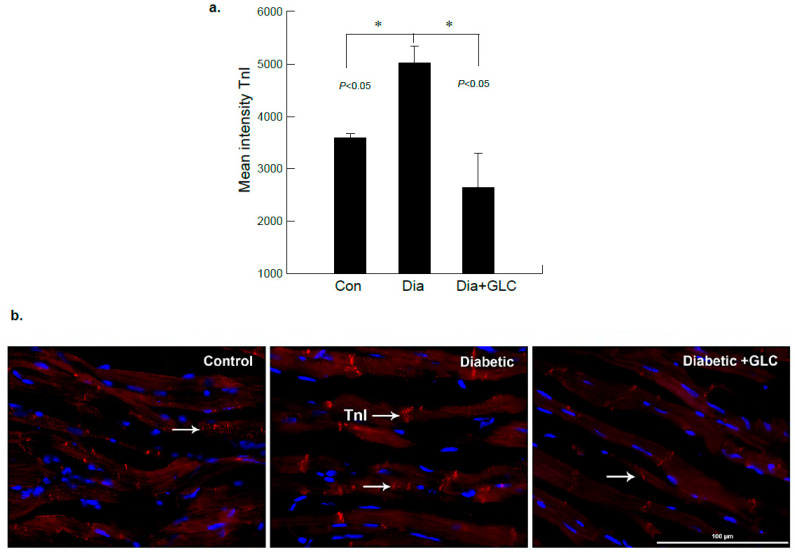
ZDF rats exhibited increased Troponin-I (TnI) expression in the cardiac tissue which was altered after GLC treatment. (**a**) Immunofluorescent images of heart tissues of lean control, ZDF diabetic and ZDF with GLC treated rats were analyzed, which showed noticeable changes in the intensity of TnI immunostaining in diabetic GLC treated rats after 4 weeks of treatment compared to ZDF diabetic rats (* *p* < 0.05). (**b**) ZDF diabetic rats demonstrated substantial increase in TnI expression compared to lean control rats as shown by representative images; arrows indicate TnI positive staining in red (Scale bar = 100 µm).

To further examine diabetes-induced cardiac damage, injury marker troponin I (TnI) was analyzed in the heart tissues from ZDF rats by immunohistochemistry. Figure 4 shows immunofluorescence images obtained from heart tissues in lean control, ZDF diabetic and ZDF animals treated with GLC. The images were analyzed and based on the intensity of the TnI immunostaining, they showed noticeable changes in diabetic GLC treated group animals at 4 weeks after treatment as compared to ZDF diabetic rats. There was also a significant increase in TnI expression in ZDF diabetic rats as compared to lean controls (Figure 4a,b).

### 3.4. Diabetic Cardiac Tissue of ZDF Rats Exhibited an Increase in the Expression of Voltage-gated Sodium Channel Na_V_1.5 and Increased Phosphorylation of p38 MAPK

**Figure 5:**
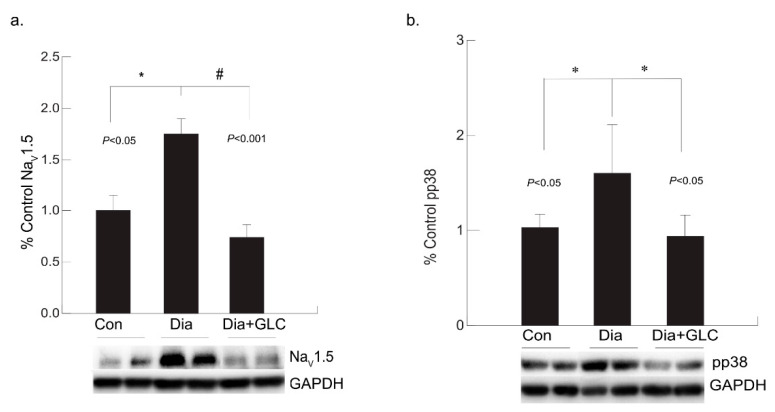
Anti-inflammatory effect of GLC reduced the increased voltage-gated sodium channel Na_V_1.5 as well as the activation of p38 MAPK in the cardiac tissue of diabetic animals. (**a**) Increased expression of Na_V_1.5 in cardiac tissue was observed in the diabetic only group of ZDF rats (* *p* < 0.05) whereas it was significantly ameliorated by 4 weeks of treatment with anti-inflammatory agent GLC (# *p* < 0.001). (**b**) p38 MAPK was greatly activated in the heart tissue under diabetic condition (* *p* < 0.05), whereas treatment with GLC in ZDF rats inhibited the increased phosphorylation of p38 MAPK (* *p* < 0.05).

Our study revealed that the expression level of Na_V_1.5 was modified in the cardiac tissue of the ZDF rats. Voltage-gated sodium channel isoform Na_V_1.5 is present in cardiac tissue which is crucial for initiation and propagation of the action potential. Increased expression of Na_V_1.5 in cardiac tissue was observed in the eighteen weeks old ZDF rats whereas it was ameliorated by treatment with anti-inflammatory agent GLC (Figure 5a). In diabetic condition, p38 mitogen-activated protein kinase (MAPK) is highly activated in many tissues including the heart and our study confirms this observation. In ZDF cardiac tissues, p38 MAPK was found to be highly phosphorylated, whereas GLC treatment inhibited the increase in the phosphorylation state of p38 MAPK (Figure 5b).

### 3.5. Hyperglycemia Mediated Expression Levels of Oxidative Stress Marker and Antioxidant Transcription Factor Were Altered with GLC Treatment in Cardiac Tissue of Type 2 Diabetic Animals

**Figure 6:**
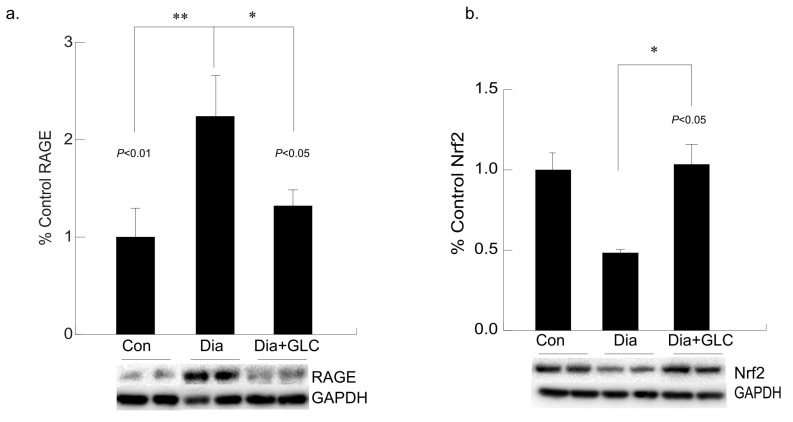
Long-term hyperglycemia exhibited altered levels of RAGE and antioxidant transcription factor, Nrf2 in ventricular cardiac tissue of diabetic rats, which were improved with GLC treatment. (**a**) Increased RAGE expression was observed in the ventricular cardiac tissue of ZDF diabetic rats compared to lean control animals (** *p* < 0.01) which was alleviated in the GLC treated diabetic animals (* *p* < 0.05). (**b**) Transcription factor Nrf2 expression in the cardiac tissues of the Type 2 diabetic animals was downregulated compared to the control animals. The ventricular cardiac tissue of ZDF animals treated with anti-inflammatory agent GLC for 4 weeks showed substantial improvement of Nrf2 expression (* *p* < 0.05).

Hyperglycemia-mediated cardiac impairment is multifaceted and elevated oxidative stress is one of the crucial contributors to this process. We observed increased expression of the receptor for advanced glycation end-products (RAGE) in the cardiac tissue of the ZDF diabetic rats compared to lean controls, whereas treatment with GLC led to a decrease in the RAGE expression in the ventricular cardiac tissues of the ZDF rats (Figure 6a). To understand whether advanced glycation end-products mediated oxidative stress in diabetes is mediated through inactivation of the antioxidant response element (ARE) signaling pathway, NF-E2-related factor 2 (Nrf2) expression was analyzed which showed a downregulation in the expression of this transcription factor in the Type 2 diabetic animals. The ventricular cardiac tissue of ZDF animals treated with anti-inflammatory agent GLC showed amelioration of Nrf2 expression (Figure 6b).

### 3.6. Alleviation of Hyperglycemia-Mediated Increase in the Expression of Fibrotic Marker TGFβ by GLC Treatment and CXCR4 Antagonist

**Figure 7:**
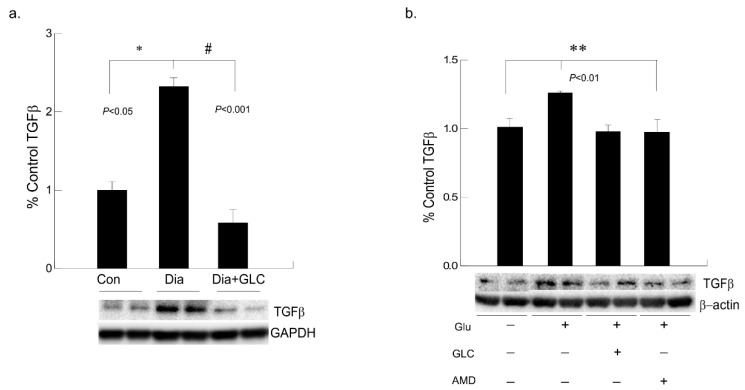
Hyperglycemic cardiac tissues and cardiomyocytes cells exhibited increased TGFβ expression which was ameliorated with CXCR4 antagonist in vitro and with GLC both in vivo and in vitro. (**a**) Fibrotic marker TGF-β was evaluated in cardiac tissue of ZDF rats which showed a marked increase compared to the lean control group (* *p* < 0.05). Four weeks of GLC treatment decreased TGFβ expression significantly in the cardiac tissue of ZDF rats (# *p* < 0.001). (**b**) Hyperglycemic AC-16 cardiomyocytes (Glu +) showed increased TGFβ expression whereas treatment with CXCR4 antagonist, AMD3100 (AMD +) showed significant decrease in TGF-β expression similar to the GLC treatment (GLC +; ** *p* < 0.01).

To understand hyperglycemia mediated cardiac fibrosis, transforming growth factor beta (TGF-β) was evaluated which showed a marked increase in the expression in the cardiac tissue of ZDF rats whereas TGF-β expression was reduced by treatment with GLC for four weeks (Figure 7a). To validate the increase in TGF-β under hyperglycemic condition and whether crosstalk exists among the TGF-β and CXCR4 pathways, AC16 cardiomyocyte cells were treated 25 mM of glucose overnight and a group of hyperglycemic cells were treated with 20 µm of CXCR4 antagonist AMD3100. Hyperglycemic cardiomyocytes showed increased TGFβ expression whereas treatment with AMD3100 showed decreased expression of TGF-β in these cells (Figure 7b). To confirm anti-inflammatory effect of GLC, cells treated with 50 µm of GLC exhibited reduced the TGF-β expression in AC16 cells pretreated 25 mM of glucose, which confirms analogous evaluation of the in vivo data.

### 3.7. Hyperglycemia Mediated Increased Expression of Chemokine Receptor CXCR4 Was Mimicked by SDF1 and Treatment with GLC Could Block the Effect in Cardiomyocytes In Vitro

**Figure 8:**
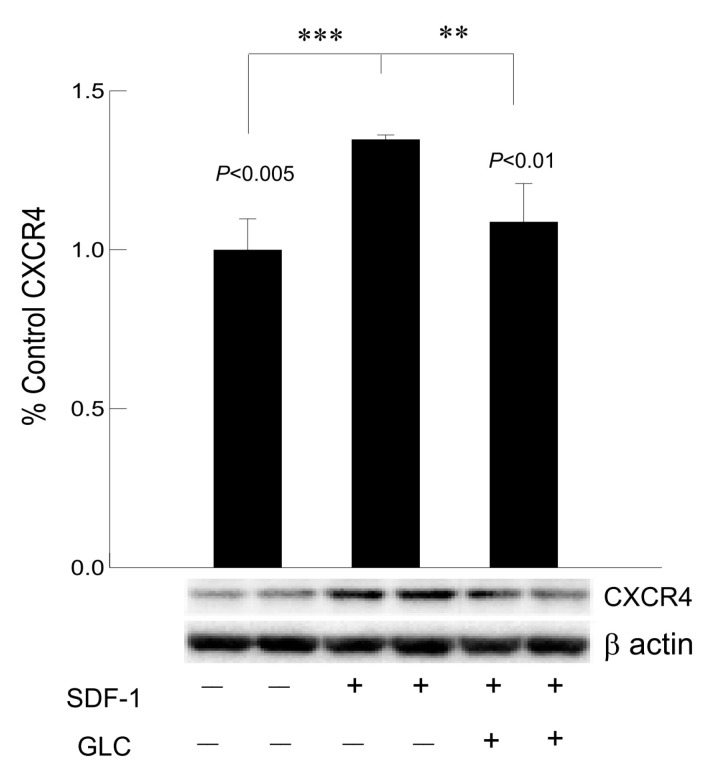
Hyperglycemia mediated increased expression of chemokine receptor CXCR4 was mimicked by SDF1 and treatment with GLC could block the effect in cardiomyocytes in vitro. Treatment with CXCR4 agonist, SDF1 showed increased expression of CXCR4 in cardiomyocyte (CM) cells in vitro (*** *p* < 0.005). CM cells were treated with GLC for 24 h showed reduction in CXCR4 expression when pre-treated with SDF1 (** *p* < 0.01).

To confirm whether GLC could block the CXCR4 expression independent of hyperglycemia, AC16 cardiomyocyte cells were treated with CXCR4 agonist SDF1 at 50 ng/mL which showed increased expression of CXCR4 in these cells (Figure 8). To confirm anti-inflammatory effect of GLC can be mediated through CXCR4 pathway, AC16 cells pretreated with SDF1 were treated with 50 µm of GLC for 24 h. The treatment with GLC exhibited reduced CXCR4 expression.

## 4. Discussion

Cardiovascular impairment is one of the major complications of long-term diabetes or uncontrolled hyperglycemia. Hyperglycemic condition in diabetes triggers non-enzymatic glycation and oxidation of proteins leading to formation of advanced glycation end products (AGEs) which plays a significant role in the development and progression of cardiovascular disease by binding its cell surface receptor (RAGE) [36]. Upregulation of RAGE has been demonstrated to be a part of pathophysiological mechanism towards cardiac dysfunction [37,38]. In this study, ventricular cardiac tissue of 18-week-old ZDF rats showed a marked increase in RAGE expression compared to age-matched lean control animals and this increase was attenuated by treatment with anti-inflammatory agent GLC. As shown by other previous studies, AGE accumulation occurs not only by oxidant stress, but also via inflammation by recruitment of inflammatory cells which may lead to vascular damage. Studies have also shown that blocking of high-mobility group box 1 (HMGB1), a proinflammatory mediator by glycyrrhizin (GLC) and knocking out of RAGE suppressed inflammation in the heart in an experimental autoimmune myocarditis mice model [39].

As a consequence of ligand binding to RAGE, initiation of various signaling pathways including p38 and SAPK/JNK MAP kinases have been demonstrated. Activation of signaling pathways increases the expression of inflammatory mediators [40,41]. Inflammatory cytokines, chemokines and their receptors play a significant role in promoting cardiac dysfunction, leading to cardiac atrophy. Hyperglycemia-induced ROS can activate p38 MAPK inducing inflammation in cardiac tissues [42] and our study showed increase in phosphorylation of p38 MAPK in the cardiac tissues of ZDF rats compared to the lean control group, whereas GLC treatment for 4 weeks considerably alleviated the phosphorylation of p38 MAPK. A number of studies have shown that p38 MAPK is significantly activated in the cardiac tissue in experimental and clinical diabetic cases whereas inhibition of p38 MAPK activation by pharmacologic agents or transgenic animals substantially prevents the development of cardiomyopathy [43], suggesting that p38 MAPK could be a novel diagnostic indicator and therapeutic target for heart diseases. 

It has been demonstrated that patients with cardiac failure have increased levels of CXCR4 expression and its ligand CXCL12/SDF1 [44]. In this study, CXCR4 levels were also higher in the heart tissues of Type 2 ZDF animals and anti-inflammatory effect of GLC can directly affect the expression of CXCR4 chemokine receptor in the diabetic heart. To confirm whether GLC could block the CXCR4 expression independent of hyperglycemic condition, AC16 cardiomyocyte (CM) cells treated with CXCR4 agonist SDF1 were analyzed. We observed an increase in expression of CXCR4 in CM cells treated with CXCR4 agonist, SDF1. To validate the anti-inflammatory effect of GLC, the cells that were pretreated with SDF1 were also treated with 50 µm of GLC for 24 h. The GLC treatment led to a reduction in CXCR4 expression in CM cells pretreated with SDF1. Earlier studies have shown that hyperglycemia could increase the expression of CXCR4 and also that CXCR4 inhibitor could reduce myocardial fibrosis in Type 1 diabetic animals [44]. Similarly, adenoviral vector mediated CXCR4 overexpression demonstrated an adverse effect on cardiomyocyte function [9]. Therefore, alleviation of increased CXCR4 expression in cardiac tissue by GLC presents a novel therapeutic target for cardiac atrophy.

Ventricular myocyte gap junction channels are composed of connexin43 (Cx43). CX43 functions as communication channel to maintain proper coupling between cardiomyocytes [3]. Earlier studies have shown that high glucose exposure for 24–48 h decreased Cx43 expression in neonatal rat cardiomyocytes [45,46]. Similar to earlier findings, our study also showed that CX43 expression was reduced in diabetic cardiac ventricular tissue. Furthermore, this study revealed that anti-inflammatory agent GLC could rescue the CX43 expression levels in diabetic heart. It is known that dysfunction of Cx43 contributes substantially to the development of cardiovascular complications in diabetic condition. In the coronary atherosclerotic and long-term diabetic patients Cx43 is downregulated which may associate with accumulation of extracellular matrix [47]. Cardiomyopathy in diabetes is frequently combined with left ventricular impairment and myocyte hypertrophy with interstitial fibrosis. As discussed earlier, impaired contractility, abnormal cellular metabolism, increased ROS as well as rise in AGEs may result in accumulation of extracellular matrix leading to cardiac fibrosis [10].

Pleiotropic cytokines such as TGFβ are one of the key contributors of cardiac fibrosis. In this study, we demonstrated that diabetes-associated hyperglycemia unequivocally activated cardiac fibroblasts and promoted excessive deposition of extracellular matrix proteins. ZDF diabetic cardiac tissue exhibited increased expression of TGFβ whereas treatment with GLC for 4 weeks alleviated TGFβ expression. Elevation of fibrogenic growth factor TGF-β may demonstrate its role in fibroblast production [48,49]. Chemokines have been described to be associated in cardiac fibrosis by the modifications in fibrogenic role of fibroblasts [50]. We have confirmed the increased expression of myocardial TGF-β in diabetic heart tissues, one of the relevant aspects that is associated with cardiac fibrosis.

Furthermore, our study demonstrated that GLC altered the SDF-1 mediated increase in chemokine receptor, CXCR4. Evidence to confirm the role for chemokine-mediated pathways in diabetes-associated cardiac fibrosis is not adequate. Our in vitro studies with AC-16 cardiomyocytes demonstrated that CXCR4 antagonist AMD3100 could block the hyperglycemia mediated increase in TGFβ similar to the GLC mediated reduction. This suggests that hyperglycemia mediated changes in CXCR4 could be involved in TGF-β induced cardiac remodeling. Further, the Gomori Trichrome (blue collagen) staining of cardiac ventricular tissue confirmed cardiac fibrosis. ZDF diabetic heart tissues exhibited increased collagen deposition with prominent connective tissue and muscle associated fibrotic changes; however, GLC treatment decreased the accumulation of blue collagen in the ZDF diabetic cardiac tissues. To validate the consequence of GLC treatment on vasoconstriction, we evaluated endothelin 1, a potent endothelium-derived vasoconstrictor peptide by Western blot analysis. Our study showed no significant change in endothelin 1 expression in diabetic heart tissues as well as its expression in the GLC-treated animals which is also supported by our earlier studies (Supplementary Materials Figure S1) [51].

Cardiac troponin T and I are important proteins that mediate the contractions of the cardiac muscles or myocardium. Troponin levels are the distinctive markers for myocardial atrophy or damage [52]. Most studies have reported that increased levels of circulating troponin are an indicator of myocardial atrophy [53]. During prolonged hyperglycemic condition, if the cardiomyocytes are irretrievably damaged, both troponin I and troponin T are highly expressed which can be detected by various measures to confirm the myocardial injury [54]. In our studies, immunohistochemical analysis showed increased expression of troponin-I in ventricular cardiac tissue of ZDF diabetic rats. GLC treatment in ZDF animals exhibited reduced expression of Troponin I in cardiac tissue sections. Hyperglycemia-induced mechanisms including increased oxidative stress, production of AGEs and activation of protein kinase C (PKC) may lead to cardiovascular complications in diabetics, resulting in increased cardiac fibrosis, hypertrophy and advancement towards severe cardiac dysfunction [55]. Increase in AGEs may induce ROS through inactivation of Nrf2. Nrf2 is an antioxidant which can protect cells against ROS through response element (ARE) signaling pathway. Nrf2 transcription factor impacts in maintenance of the oxidative homeostasis [56]. Long-term diabetes impairs this antioxidant capability of the heart. Our study suggests that the anti-inflammatory effect of GLC treatment could block activation of AGEs through decreased expression of RAGE, which eventually increases Nrf2 expression.

Voltage gated sodium channel Na_V_1.5 is highly specific to cardiomyocytes which are responsible for initiation and propagation of action potential in the heart. Small variations in subcellular organization at the intercalated discs, T-tubules and lateral membranes of this channel may affect the overall physiology and electrical activity [57]. Na_V_1.5 exhibits cardiac tissue specific expression pattern at the transcriptional level. Post-translational modification of the channel is also highly regulated towards its expression and propagation of action potentials. Additionally, changes in the expression level of Na_V_1.5 as well as the current density were often observed in cardiac failure. Na_V_1.5 is post-translationally modified, anchored to the cytoskeleton and eventually it is endocytosed and degraded. Our study demonstrates a significant increase in Na_V_1.5 expression in the ventricular diabetic heart tissue of the ZDF rats, suggesting that there could be alteration in glycosylation or changes in protein turnover which may contribute to the increased expression of Na_V_1.5 in diabetic animals compared to lean controls. Furthermore, treatment with GLC modified the channel expression in diabetic heart. Attenuation of cardiac fibrotic remodeling by GLC may indicate a better therapeutic opportunity towards hyperglycemia mediated myocardial atrophy in diabetics.

## 5. Conclusions

Hyperglycemic condition in diabetes can elevate oxidative stress and inflammation that may result in cardiac remodeling and may lead to hypertrophy of the myocardium. In this study, diabetic ZDF cardiac tissue exhibited increase in inflammation and fibrosis that could lead to cardiac damage. Therefore, it is crucial to decrease oxidative stress and inflammation to avoid further cardiac impairment. Our study demonstrated that glycyrrhizin exhibits anti-inflammatory effect on diabetic cardiac tissue and that it can prevent cardiac damage by altering oxidative stress and inflammation. Further research is required to unveil specific inflammatory markers for cardiac dysfunction in diabetes and to better understand the comprehensive cellular and molecular mechanisms associated with diabetic cardiomyopathy.

## Data Availability

All data generated or analyzed during the current study are included in this published article.

## Supplementary Materials

The following are available online at https://www.mdpi.com/article/10.3390/biom11040569/s1, Supplementary Materials Figure S1. No significant change in endothelin 1 expression in diabetic heart tissues as well as its expression in the GLC-treated animals. Supplementary Materials Figure S2. No significant change in blood glucose levels and body weight in diabetic GLC-treated animals as shown earlier (Supplementary Materials Figure S4; https://doi.org/10.3390/ijms21030881). Supplementary Materials Figure S3. Images showing changes in collagen deposition (in blue) in ZDF type 2 diabetic, diabetic with GLC treatment and lean control rats.

## Abbreviations

CXCR4	Chemokine C-X-C motif receptor 4
RAGE	Receptor for advanced glycation end products
ZDF	Zucker diabetic fatty
GLC	Glycyrrhizin
TGF-β	Transforming growth factor beta
CX43	Connexin-43
Nrf2	Nuclear factor erythroid-2-related factor 2
TnI	Troponin I
