# Supplementary material for: Cardioprotective Effect of Glycyrrhizin on Myocardial Remodeling in Diabetic Rats
Source: Biomolecules. 2021 Apr 13;11(4):569. doi: 10.3390/biom11040569 (PMC8069839; doi:10.3390/biom11040569)
Supplement: Supplementary file 1 [file biomolecules-11-00569-s001.pdf]

biomolecules

Supplementary Materials:

Suppl. Fig. 1

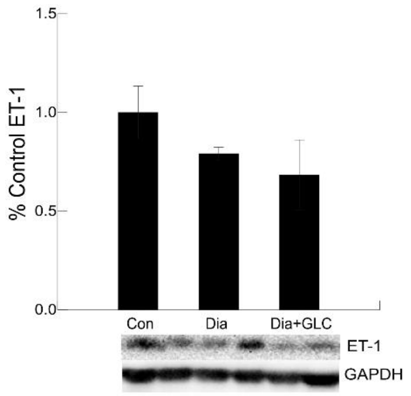

Suppl. Fig. 1. No significant change in endothelin 1 expression in diabetic heart tissues as well as its expression in the GLC-treated animals.

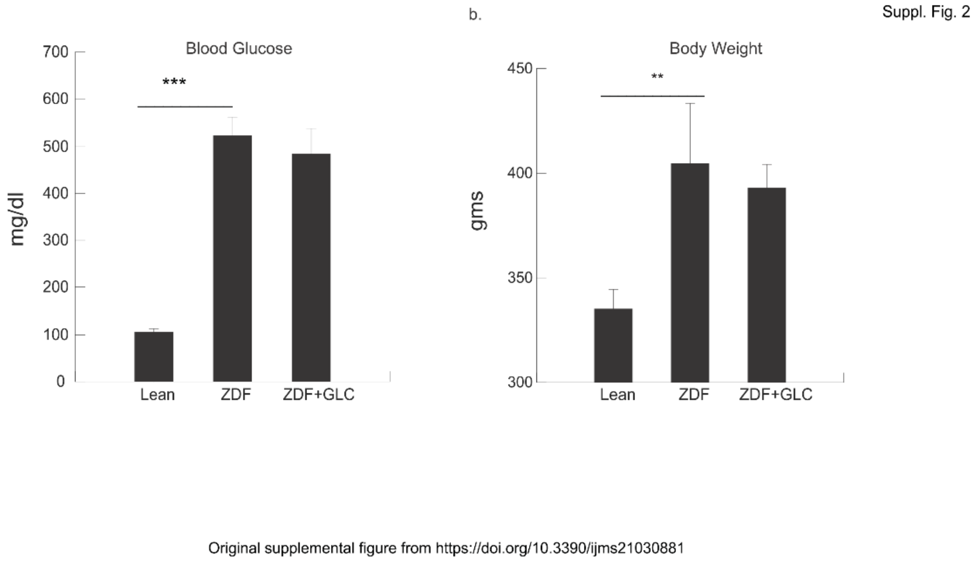

Suppl. Fig. 2. No significant change in blood glucose levels and body weight in diabetic GLC-treated animals as shown earlier (Fig S4; https://doi.org/10.3390/ijms21030881).

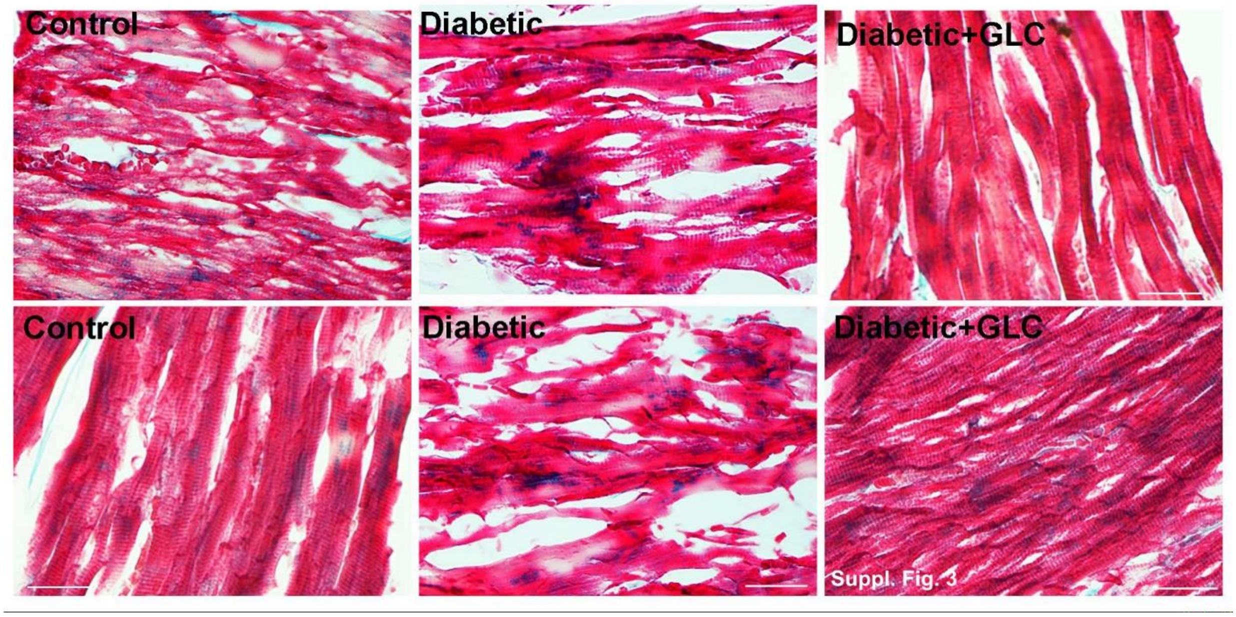

Suppl. Fig. 3. Images showing changes in collagen deposition (in blue) in ZDF type 2 diabetic, diabetic with GLC treatment and lean control rats.
